# Data Mining Strategies for Understanding Birth Patterns in Dairy Cattle: Single and Multiple Birth Analysis

**DOI:** 10.1002/vms3.70890

**Published:** 2026-03-25

**Authors:** Mostafa Ghaderi‐Zefrehei, Maryam Montazeri‐Najafabadi, Farjad Rafeie, Effat Nasre Esfahani, Mohammad Reza Abbaszadeh Bavi Soflaei, Mojtaba Kafi, Mohammad Hossein Banabazi

**Affiliations:** ^1^ Department of Animal Science Agricultural Faculty Yasouj University Yasouj Iran; ^2^ Department of Animal Science Faculty of Agricultural Sciences University of Guilan Rasht Iran; ^3^ Department of Agricultural Biotechnology Faculty of Agricultural Sciences University of Guilan Rasht Iran; ^4^ Department of Agriculture Payame Noor University Tehran Iran; ^5^ Department of Computer Engineering University College of Nabi Akram Tabriz Iran; ^6^ Department of Animal Reproduction School of Veterinary Medicine Shiraz University Shiraz Iran; ^7^ Department of Animal Biosciences (HBIO) Swedish University of Agricultural Sciences (SLU) Uppsala Sweden

**Keywords:** algorithms, dairy cow, data mining, multiple birth, Waikato Environment for Knowledge Analysis (WEKA)

## Abstract

Data mining is a robust methodology designed to predict feature performance, identify trends within datasets, classify data based on similarities and optimize resource allocation. In the context of dairy farming, multiple birth is generally regarded as an undesirable outcome due to its adverse effects on cow fertility rates and the health of multiple calves, ultimately impacting farm profitability. This study conducts a comprehensive evaluation of various machine learning algorithms for the classification of multiple birth data using the Waikato Environment for Knowledge Analysis (WEKA) platform. The performance of 21 algorithms was evaluated using multiple metrics, including mean absolute error (MAE), root mean square error (RMSE), Kappa statistic, classification accuracy and computational time. The results reveal significant variability in the performance of the algorithms. Notably, the radial basis function (RBF) and support vector machine (SVM) algorithms outperformed others, achieving the lowest MAE and RMSE values, along with the highest Kappa statistics and classification accuracy on both training and test datasets. Specifically, the RBF model achieved a perfect Kappa statistic of 1 on the training set, indicating flawless agreement between predicted and actual classifications. In contrast, algorithms such as Voted Perceptron and Simple Cart exhibited higher error rates and lower classification accuracy, rendering them less suitable for this task. The analysis also highlighted trade‐offs between accuracy and computational efficiency, with algorithms like *K*‐nearest neighbours (*K*NN) and Random Forest providing a balanced performance. These findings emphasize the critical importance of selecting appropriate algorithms and evaluation metrics to achieve robust and reliable classification outcomes in multiple birth data analysis.

## Graphical abstract

Multiple machine learning algorithms are evaluated to predict multiple birth in dairy cattle using classification techniques. Among 21 models, radial basis function (RBF) and support vector machine (SVM) demonstrate superior performance with the highest accuracy and lowest error rates. The findings highlight the importance of model selection for improving reproductive management and optimizing productivity in dairy farming systems.

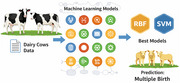

## 1 Introduction

The analysis of dairy cattle data has a well‐documented history, with various classification techniques playing a pivotal role (Adamczyk et al. 2016; Zaborski et al. 2016). Among these, artificial neural networks (ANNs) have gained prominence due to their versatility in analysing mastitis, milk production and genetic evaluation. ANNs have also been extensively applied in oestrus detection, hardiness assessment, lameness research and nutritional studies in dairy cattle (Nielen et al. 1989, 1995; Cortes and Vapnik 1995; Lacroix et al. 1997; Salehi et al. 1999; Yang et al. 1999, 2000; Sanzogni and Kerr 2001; Grzesiak et al. 2003; Pastell and Kujala 2007; Hosseinnia et al. 2007; Cavero et al. 2008; Chen et al. 2008, 2009; Njubi et al. 2009; Hassan et al. 2009; Grzesiak et al. 2010; Zaborski and Grzesiak 2011a, 2011b; Gorgulu 2012; Shahinfar et al. 2012; Sumon Shahriar et al. 2016). Furthermore, capillary microfluidic sensors have been employed to accurately identify the most fertile period in dairy cows (Boreckia et al. 2010). ANNs have also been utilized as a computational tool for detecting durability traits in dairy cattle, leveraging advanced classification techniques and predictive models (Morrison et al. 1985; Piwczyński et al. 2013; Zaborski et al. 2014). Multiple birth in cattle has been a subject of research since the early 1900s (Johansson et al. 1974). Although approximately 10% of dairy cattle experience multiple birth during pregnancy, beef cows exhibit a significantly lower incidence, typically ranging from 1% to 4% (Komisarek and Dorynek 2002; Cabrera and Fricke 2021). Multiple pregnancies in dairy cows are generally considered undesirable due to their association with various reproductive disorders, including dystocia, perinatal mortality and metabolic diseases, all of which contribute to substantial economic losses for dairy farms (Cabrera and Fricke 2021; Fricke 2001). The negative impacts of multiple birth extend to the productive lifespan of cows, with those calving multiple exhibiting a significantly shorter lifespan compared to those delivering singletons (López‐Gatius 2020; Garcia‐Ispierto and López‐Gatius 2021). Complications such as placental abruption, dystocia and metritis often lead to severe outcomes, including retained placenta, uterine infections and repeat breeding syndrome (Gregory et al. 1996; Echternkamp and Gregory 1999). Multiple birth also adversely affects maternal health, and in 92% of cases where the calves are male and female, the female calf is rendered sterile due to freemartinism (Buoen et al. 1992). The incidence of multiple birth increases with parity, with higher rates observed in cows that have calved more than three times (Garcia‐Ispierto and López‐Gatius 2018). Parity and milk production levels are critical factors predisposing dairy cattle to multiple ovulation and multiple birth. Studies have demonstrated that higher parity correlates with an increased probability of multiple ovulations, potentially resulting in multiple birth (Fitzgerald et al. 2014; Macmillan et al. 2018; McGovern et al. 2021). Additionally, elevated milk production has been linked to higher rates of multiple ovulations, attributed to hormonal changes during lactation (Wiltbank et al. 2000; López‐Gatius et al. 2005). These factors collectively influence the reproductive performance and health of dairy cows, underscoring their importance in dairy management practices.

The economic losses associated with multiple birth range from 59 to 161 per pregnancy, driven by factors such as reduced milk production, increased incidence of dystocia, perinatal mortality, metabolic diseases, decreased fertility and higher culling rates (Mur‐Novales et al. 2018; Cabrera and Fricke 2021). A comprehensive economic model estimates the average total loss per multiple birth at 108.51, accounting for costs related to reduced milk yield, increased culling and additional veterinary care (Beerepoot et al. 1992). Furthermore, the overall negative economic impact of multiple birth on dairy farm profitability in the United States is estimated at approximately 108.51, accounting for costs related to reduced milk yield, increased culling and additional veterinary care (Beerepoot et al. 1992). Furthermore, the overall negative economic impact of multiple birth on dairy farm profitability in the United States is estimated at approximately 96 million annually (Mur‐Novales et al. 2018). Management strategies, such as early diagnosis and manual embryo reduction, can mitigate these losses, potentially reducing economic impacts by 23–45 per multiple birth (Cabrera and Fricke 2021).

This study aims to address the challenges posed by multiple birth in dairy cattle through the application of machine learning classifiers. The primary objective is to evaluate various machine learning models to predict multiple birth occurrences by analysing factors influencing multiple birth rates. Previous research has established that multiple birth is a multifactorial trait influenced by genetics, parity and milk production levels (Fricke 2001; López‐Gatius 2020). This research seeks to leverage machine learning techniques to uncover patterns and relationships that may elude conventional statistical approaches. Additionally, the study aims to identify the most efficient predictive model to assist dairy farmers in controlling and minimizing multiple birth rates. This is critical for enhancing both profitability and animal welfare, as elevated multiple birth rates are associated with a higher likelihood of reproductive complications, increased operational costs and a shortened productive lifespan of cows (Cabrera and Fricke 2021; López‐Gatius et al. 2023). The findings of this study have the potential to inform better breeding decisions and management practices, ultimately contributing to the sustainability of dairy operations.

## 2 Materials and Methods

### 2.1 Dataset Description

The dataset used in this study pertains to FOKA Holstein dairy cows herd located in Isfahan FOKA stands as one of the largest and most sophisticated dairy farms in the Middle East (Ghaderi‐Zefrehei et al. 2016). It accommodates around 12,500 animals and generates approximately 60,000 tons of milk each year. The farm achieves an average daily yield of 44 kg of milk per cow, utilizing state‐of‐the‐art management techniques, such as total mixed ration (TMR) feeding and advanced health monitoring systems. Additionally, FOKA is actively involved in research aimed at improving livestock productivity and plays a significant role in Iran's dairy sector, fostering local employment and enhancing the quality of milk for both domestic consumption and potential export markets. The information presented in Tables 1 and 2 highlights the frequency of calving occurrences, categorized as a binary trait: Code 1 denotes single births, whereas Code 2 represents multiple birth and higher order multiples. Table 3 presents the distribution of multiple birth data, which serves as the dependent variable in this research. To prepare the dataset, SQL codes were implemented using Microsoft Access. Given the critical role of feature selection in prediction accuracy, redundant or irrelevant features were identified and removed to enhance the analysis. The dataset was partitioned into training and testing sets using Waikato Environment for Knowledge Analysis (WEKA), with 70% allocated for training and 30% for testing. This partitioning ensures robust model evaluation and generalizability.

**TABLE 1:** List of quantitative variables/features/attributes/predictors used in this study.

Variable/Feature/Attribute/Predictor	Mean	Standard deviation	Range
Length of gestation (day)	276.2	5.7	252.0–290.0
Calving age (day)	1502.7	458.3	1096.0–3141.0
Open days (day)	211.0	76.1	121.0–461.0
Calving interval (day)	487.2	76.8	387.0–742.0
Adjusted milk (ME‐305‐2×) (kg)	9304.66	1512.96	4372.49–15,356.19
Adjusted fat (ME‐305‐2×) (kg)	260.76	61.67	85.17–423.86
Adjusted protein (ME‐305‐2×) (kg)	214.583	77	19.73–429.43
Dry off period (day)	66.3	24.1	31.0–198.0
Adjusted milk (ME‐305‐2×) (mother) (kg)	8544.63	1108.63	4961.19–12,512.43
Adjusted fat (ME‐305‐2×) (mother) (kg)	2.96	0.32	1.58–4.35
Adjusted protein (ME‐305‐2×) (mother) (kg)	1.960	0.410	1.031–3.300
Inbred cow (%)	0.018	0.011	0.000–0.046

**TABLE 2:** Descriptive list of qualitative variables/features/attributes/predictors used in this study.

Trait	Description	Mean of function
Calving ease	The ease with which a cow gives birth, often assessed by the need for assistance during delivery. It is a significant trait in breeding programmes for dairy cattle to reduce complications and improve animal welfare (Eaglen et al. 2011)	4
Year of calving	The specific year in which a cow gives birth, important for tracking reproductive performance and management decisions in livestock (Eaglen et al. 2010)	24
Type of parturition	Refers to the method of delivery, which can be natural (vaginal) or assisted (e.g., caesarean section), impacting both maternal and neonatal health outcomes (Wood 1999)	3
Duration of placenta	The time the placenta remains attached post‐delivery, which can affect postpartum recovery and health of the cow (Hur et al. 2011)	2
Ovarian and uterine diseases	Conditions affecting the ovaries and uterus that can impact fertility and overall reproductive health in animals (Iraha et al. 2017)	2
Number of abortions	The count of pregnancy losses occurring within a specified timeframe, used to assess reproductive performance and health (Ramer et al. 2024)	3
Number of generations	The count of successive generations in a breeding programme, important for genetic diversity and selection (Hutchison et al. 2017)	22–30
Number of insemination leading to pregnancy	The total number of artificial insemination attempts that result in successful pregnancies, indicating fertility efficiency (Inchaisri et al. 2011)	1–10
Season of insemination	The specific time of year when artificial insemination occurs, which can influence conception rates due to environmental factors (Muller et al. 2014)	4
Parity number	The number of times a dairy cow has given birth. It is a critical factor influencing various aspects of dairy production, including milk yield, reproductive performance and health status (Liu et al. 2024)	6
Age at first calving	The age at which a heifer gives birth for the first time, impacting lifetime productivity and reproductive efficiency (Berry and Cromie 2009)	24
Season of insemination	The time of year when an inoculation (e.g., vaccination or artificial insemination) is administered, which can affect the immune response or reproductive outcomes (Linder et al. 2011)	4
Service per conception	The total count of successful pregnancies achieved through artificial insemination within a specific timeframe (Rutten et al. 2016)	13
Ovarian diseases	Disorders affecting the ovaries, which can lead to infertility or other reproductive issues (Arlt and Haimerl 2016)	2
Durability	In a biological context, refers to the process of tissue or material becoming more rigid or resistant, often related to physiological changes during pregnancy or development (Holschemacher 2004)	4
Type of birth	The classification of birth method, such as vaginal or caesarean, which can influence maternal and neonatal health outcomes (Walrath 2003)	3
Dystocia	A condition of difficult labour or delivery, often requiring medical intervention, and can be classified based on severity and causes (Neal et al. 2015)	4

**TABLE 3:** Distribution of birth status (dependent/output variable).

Birth status	Number	Percent
Single birth	691	56.51
Multiple birth	531	43.48

### 2.2 Classification Techniques

**FIGURE 1:**
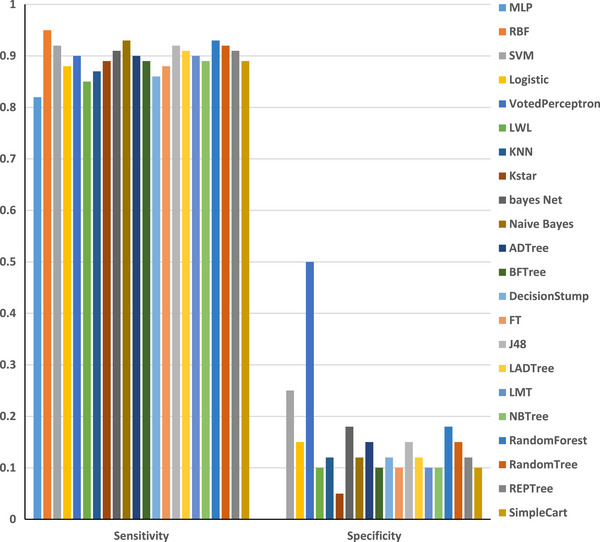
Comparison of the performance of various algorithms in terms of sensitivity and specificity. *K*NN, *K*‐nearest neighbour; MLP, Multilayer Perceptron; RBF, Radial Basis Function; SVM, support vector machine.

**FIGURE 2:**
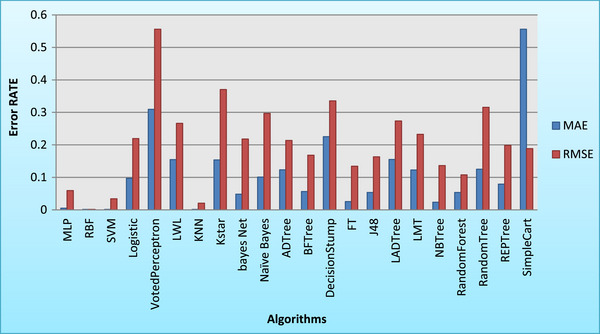
Comparison of the performance of various algorithms in terms of MAE and RMSE in the training dataset. *K*NN, *K*‐nearest neighbour; MAE, mean absolute error; MLP, Multilayer Perceptron; RBF, Radial Basis Function; RMSE, root mean square error; SVM, support vector machine.

**FIGURE 3:**
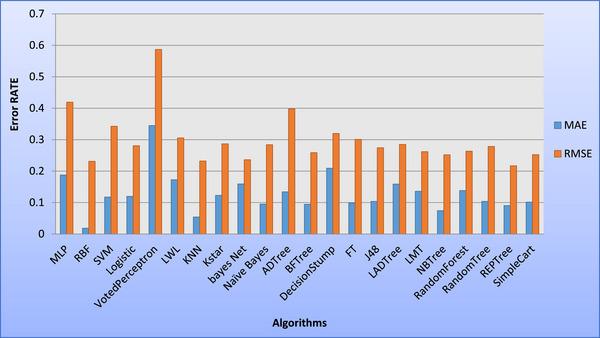
Comparison of the performance of various algorithms in terms of MAE and RMSE in the test dataset. *K*NN, *K*‐nearest neighbour; MAE, mean absolute error; MLP, Multilayer Perceptron; RBF, Radial Basis Function; RMSE, root mean square error; SVM, support vector machine.

In this study, root mean square error (RMSE) and mean absolute error (MAE) were employed as primary metrics to evaluate the performance of machine learning algorithms for multiple birth data classification. RMSE, a widely used measure of prediction accuracy, penalizes larger errors more significantly, providing a robust assessment of model performance. MAE, on the other hand, represents the average absolute difference between predicted and actual values, offering a straightforward interpretation of error magnitude. These metrics were calculated using established formula: RMSE = $\sqrt{\frac{\sum_{i=1}^{n}{\left(y_{i,\mathrm{predicted}}-y_{i,\mathrm{actual}}\right)}^{2}}{n}}$ and MAE = $\frac{1}{n}\sum_{i=1}^{n}\left|y_{i,\mathrm{predicted}}-y_{i,\mathrm{actual}}\right|$, where $y_{i,\mathrm{predicted}}$ is the predicted value for the *i*th observation, $y_{i,\mathrm{actual}}$ is the actual value for the *i*th observation, and *n* is the total number of observations (Gregory et al. 1996; Echternkamp and Gregory 1999; Garcia‐Ispierto and López‐Gatius 2021). WEKA, a machine learning toolkit developed by Waikato University in New Zealand, was utilized for data analysis. WEKA, compatible with various operating systems and available under the GNU Public License, offers a comprehensive suite of algorithms for data mining tasks. Feature selection algorithms were applied to the formatted data (Table 4) to improve the efficiency of the analysis. Multiple models were trained to predict multiple birth outcomes, and their performance was evaluated using metrics such as MAE, RMSE, Kappa statistic, correctly classified instances (in percentage and value), incorrectly classified instances (in percentage and value) and time taken (in seconds). This comprehensive evaluation framework ensures a thorough comparison of model performance. Additionally, sensitivity and specificity statistics for the evaluated models are illustrated in Figure 1, whereas Figures 2 and 3 depict the training error rates for the prediction tasks.

**TABLE 4:** Results of the neural network models.

Model	Mean absolute error	Root mean square	Kappa static (%)	Correctly classified instances (%)	Incorrectly classified instances (%)	Time taken (s)
MLP	0.1877	0.4194	0.8475	81.4208	6.5745	63.74
RBF	0.0186	0.2313	0.6207	87.7049	18.5792	1.45

Abbreviations: MLP, Multilayer Perceptron; RBF, Radial Basis Function.

### 2.3 Performance Metrics

In binary classification tasks, machine learning classifiers assign a numerical score between 0 and 1 to indicate the likelihood of an instance belonging to a specific class (e.g., ‘single‐born’ as the target class). By comparing this score to a predefined threshold, the classifier determines the final prediction. The predictions can result in four outcomes: true positive (TP), true negative (TN), false positive (FP) and false negative (FN).

Key evaluation metrics include precision and recall. Precision measures the proportion of TP predictions among all positive predictions, whereas recall quantifies the proportion of TP predictions among all actual positive instances. The confusion matrix, a tabular representation of model predictions versus actual labels, provides a clear visualization of model performance across different classes.

Before delving into the evaluation metrics, it is essential to define the following terms:

- TP: Instances correctly predicted as positive.
- TN: Instances correctly predicted as negative.
- FP: Instances incorrectly predicted as positive.
- FN: Instances incorrectly predicted as negative.

#### 2.3.1 Accuracy

Accuracy measures the proportion of correctly predicted instances (both TPs and TNs) out of the total number of instances. It is calculated as

(1) $$\mathrm{Accuracy}=\frac{\mathrm{Number}\;\mathrm{of}\;\mathrm{correct}\;\mathrm{predictions}}{\mathrm{Total}\;\mathrm{number}\;\mathrm{of}\;\mathrm{predictions}}$$

#### 2.3.2 Precision

Precision evaluates the quality of positive predictions by calculating the proportion of TPs among all predicted positives. A higher precision indicates fewer FPs:

(2) $$\mathrm{Precision}=\frac{\mathrm{True}\;\mathrm{positives}}{\mathrm{True}\;\mathrm{positives}+\mathrm{False}\;\mathrm{positives}}$$

#### 2.3.3 Recall

Recall, also known as sensitivity or TP rate, measures the proportion of actual positives correctly identified by the model. A higher recall indicates fewer FNs:

(3) $$\mathrm{Recall}=\frac{\mathrm{True}\;\mathrm{positives}}{\mathrm{True}\;\mathrm{positives}+\mathrm{False}\;\mathrm{negatives}}$$

#### 2.3.4 *F*1‐Score

The *F*1‐Score, the harmonic mean of precision and recall, provides a balanced evaluation of model performance, particularly in imbalanced datasets:

(4) $$F1=2\times \;\frac{\mathrm{Precision}\times \;\mathrm{Recall}}{\mathrm{Precision}+\mathrm{Recall}}$$

#### 2.3.5 Receiver Operating Characteristic (ROC)/Area Under the Curve (AUC)

The ROC curve illustrates the trade‐off between TP rate (recall) and FP rate (FPR) across different thresholds. The AUC provides a summary of the model's overall performance.

#### 2.3.6 Likelihood Ratio Positive (LR+)

The LR+ measures the effectiveness of a test in identifying TPs relative to FPs. A higher LR+ indicates better diagnostic performance:

(5) $$\left(\mathrm{LR}+\right)=\frac{\mathrm{True}\;\mathrm{positive}\;\mathrm{rate}}{1-\mathrm{True}\;\mathrm{negative}\;\mathrm{rate}}$$

#### 2.3.7 False Negative Rate (FNR)

The FNR quantifies the proportion of actual positives incorrectly predicted as negatives. A lower FNR indicates better model performance:

(6) $$\mathrm{FNR}=\frac{\mathrm{False}\;\mathrm{negative}\;\mathrm{rate}}{\mathrm{False}\;\mathrm{negative}\;\mathrm{rate}+\mathrm{True}\;\mathrm{positive}\;\mathrm{rate}}$$

#### 2.3.8 Positive Predictive Value (PPV)

PPV, equivalent to precision, measures the proportion of TPs among all predicted positives:

(7) $$\mathrm{PPV}=\frac{\mathrm{True}\;\mathrm{positive}\;\mathrm{rate}}{\mathrm{False}\;\mathrm{positive}\;\mathrm{rate}+\mathrm{True}\;\mathrm{positive}\;\mathrm{rate}}$$

#### 2.3.9 Geometric Mean (GM)

The GM balances sensitivity and specificity, providing a robust performance measure for imbalanced datasets:

(8) $$G-\mathrm{Mean}=\sqrt{\mathrm{Sensitivity}\;\times \mathrm{Specificity}}$$

#### 2.3.10 Jaccard Index

The Jaccard Index measures the similarity between predicted and actual classes, calculated as

(9) $$\mathrm{Jaccard}=\frac{\mathrm{True}\;\mathrm{positive}\;\mathrm{rate}}{\mathrm{True}\;\mathrm{positive}\;+\mathrm{False}\;\mathrm{positive}\;+\mathrm{False}\;\mathrm{negative}}$$

#### 2.3.11 Balanced Classification Rate (BCR)

BCR, the arithmetic mean of sensitivity and specificity, provides a balanced evaluation of model performance:

(10) $$\mathrm{BCR}=\frac{\mathrm{True}\;\mathrm{positive}\;\mathrm{rate}+\mathrm{True}\;\mathrm{negative}\;\mathrm{rate}}{2}$$

## 3 Results

The performance of various models was evaluated using classification metrics, including accuracy (classification rate), MAE, RMSE and related techniques. The results are systematically presented in Tables 4, 5, 6, 7, 8, 9.

**TABLE 5:** Results of the support vector machine (SVM).

Model	Mean absolute error	Root mean square	Kappa static (%)	Correctly classified instances (%)	Incorrectly classified instances (%)	Time taken (s)
SVM	0.1175	0.3428	0.7571	88.2514	11.7486	0.44

Abbreviation: SVM, support vector machine.

**TABLE 6:** Results of the Logistic Regression model.

Model	Mean absolute error	Root mean square	Kappa static (%)	Correctly classified instances (%)	Incorrectly classified instances (%)	Time taken (s)
Logistic Regression	0.1198	0.2806	0.8168	90.9836	9.0164	0.39

**TABLE 7:** Results of the Voted Perceptron, Random Forest and Random Tree models.

Model	Mean absolute error	Root mean square	Kappa static (%)	Correctly classified instances (%)	Incorrectly classified instances (%)	Time taken (s)
Voted Perceptron	0.3451	0.5869	0.264	65.5738	34.4262	0.25
Random Forest	0.1383	0.2635	0.8942	88.2514	5.1913	0.13
Random Tree	0.1036	0.2784	0.8332	91.8033	8.1967	0.03

**TABLE 8:** Results of the *K*‐nearest neighbours (*K*NN) and Kstar models.

Model	Mean absolute error	Root mean square	Kappa static (%)	Correctly classified instances (%)	Incorrectly classified instances (%)	Time taken (s)
*K*NN	0.0543	0.2323	0.6775	84.153	15.847	0.08
Kstar	0.1232	0.2866	0.0808	84.0638	17.9362	0

**TABLE 9:** Results of the Bayesian models.

Model	Mean absolute error	Root mean square	Kappa static (%)	Correctly classified instances (%)	Incorrectly classified instances (%)	Time taken (s)
Bayes Net	0.1593	0.2364	0.8884	94.5355	5.4645	0.24
Naïve Bayes	0.0949	0.2838	0.804	90.4372	9.5628	0.09
NB Tree	0.0746	0.2519	0.8559	92.8962	7.1038	3.84

### 3.1 Neural Networks

This section discusses the performance of neural network models, specifically the Multilayer Perceptron (MLP) and the Radial Basis Function Network (RBF). Detailed performance metrics for these models are provided in Table 4.

### 3.2 Support Vector Machines (SVM)

The results of the SVM model are presented in this section. A comprehensive summary of its performance metrics is available in Table 5.

### 3.3 Linear Models

This section analyses the performance of linear models, with a particular focus on Logistic Regression. The detailed performance metrics for this model are outlined in Table 6.

### 3.4 Ensemble Models

The performance of ensemble methods, including the Voted Perceptron, Random Forest and Random Tree models, is examined in this section. The corresponding performance metrics are detailed in Table 7.

### 3.5 Instance‐Based Models

This section explores the performance of instance‐based methods, particularly the *K*‐nearest neighbours (*K*NN) and Kstar models. The performance metrics for these models are summarized in Table 8.

### 3.6 Bayesian Models

The performance of Bayesian models, including Bayes Net, Naïve Bayes and NB Tree, is analysed in this section. Detailed performance metrics are provided in Table 9.

### 3.7 Accuracies

The preceding section discussed the training results, with a focus on the accuracies achieved by the various prediction models. This section provides insights into the models' performance during the training phase, highlighting their ability to learn from the provided data (Table 10). By examining the accuracies attained by each model, we gain a deeper understanding of their efficacy and potential for real‐world applications.

**TABLE 10:** Accuracies of various machine learning models.

Models	Train accuracy	Test accuracy
MLP	99.6491	81.4208
RBF	100	87.7049
SVM	99.883	88.2514
Logistic Regression	93.5673	90.9836
Voted Perceptron	69.0058	65.5738
LWL	91.8129	88.7978
*K*NN	100	84.153
Kstar	85.2459	84.0638
Bayes Net	95.2047	94.5355
Naïve Bayes	89.9415	90.4372
Random Forest	99.4152	88.2514
Random Tree	90.0585	91.8033

Abbreviations: *K*NN, *K*‐nearest neighbour; MLP, Multilayer Perceptron; RBF, Radial Basis Function; SVM, support vector machine.

## 4 Discussion

The analysis revealed a sensitivity of 93% compared to a specificity of 69%, indicating that the identification of cows with multiple birth was more accurate relative to the overall multiple birth population. Consequently, the number of single births was lower than the total number of single cow entries. The model demonstrated optimal precision (Adamczyk et al. 2016), with complete confidence in predicting multiple birth cows, as they were accurately identified with a 100% probability. The negative predictive value (NPV) was 0.8, suggesting an 80% chance that a cow predicted to have breeding potential would produce a calf. In contrast to the FPR, the FNR was elevated, indicating that a higher number of multiple birth cows were misclassified. The RBF and *K*NN models exhibited the highest resolution for multiple birth values, achieving an AUC of 1. The SVM and MLP models also demonstrated strong performance, with AUC values exceeding 98%. These near‐perfect scores indicate that the predicted values closely align with the actual values, resulting in highly accurate predictions. A study by Kumar and Sharma (2014) investigated the correlation between single nucleotide polymorphisms (SNPs), multiple birth rates and ovulation rates in a genetically diverse population selected for their propensity to conceive multiple birth. Neural networks were found to be highly effective in this context (Njubi et al. 2009). Additionally, milk production and lifespan were studied using multi‐layered neural networks to train the system (Kumar and Sharma 2014). Research on Holstein cows revealed that parity, season and age at first calving significantly influenced multiple birth rates (Mahani et al. 2016). Another study identified breed, season, parity number and milk yield as the most significant factors associated with multiple birth (Fricke 2001). Parity number and gestation length were also found to be critical variables affecting multiple birth rates (Cady and Van Vleck 1978). Other factors influencing multiple birth in dairy cows include breed (Cady and Van Vleck 1978; Anderson et al. 1982; Nielen et al. 1989; Ryan and Boland 1991), inbreeding (Gregory et al. 1990; John and Langley 2013; Gáspárdy et al. 2018), ovarian–uterine diseases (Labhsetwar et al. 1963), parity number (Labhsetwar et al. 1963; Syrstad 1974; Johansson et al. 1974; Cady and Van Vleck 1978; Anderson et al. 1982; Gregory et al. 1990; Nielen et al. 1995; Gáspárdy et al. 2018), season (Nielen et al. 1989; Gregory et al. 1990; Ryan and Boland 1991; Gáspárdy et al. 2018), milk production or peak milk yield (Syrstad 1974; Fricke 2001), previous multiple birth rates (Nielen et al. 1989; Fricke 2001), hormonal and pharmaceutical interventions (e.g., GnRH, PGF2a and antibiotics) (Syrstad 1974; Kinsel et al. 1998; Gáspárdy et al. 2018) and heifer body weight (Koong et al. 1982; Ryan and Boland 1991). As illustrated in Figure 1, the classification of multiple birth data using various algorithms revealed significant variability in sensitivity and specificity metrics. The RBF model demonstrated a balanced performance, achieving high values in both sensitivity and specificity. In contrast, algorithms such as MLP, Logistic Regression and tree‐based methods (e.g., J48 and Random Forest) exhibited high sensitivity but considerably lower specificity. This disparity suggests that although these algorithms are effective in identifying positive cases, they struggle with accurately classifying negative cases. Furthermore, significant variability in error rates was observed across different models. For both training and test datasets, algorithms such as MLP, RBF, SVM and Logistic Regression consistently exhibited low MAE and RMSE values, indicating robust performance and strong generalization capabilities. Conversely, the Voted Perceptron algorithm displayed the highest error rates in both datasets, suggesting its inadequacy for this classification task. The Simple Cart algorithm also demonstrated relatively high error rates (see Figures 2 and 3). The consistency in performance trends between the training and test datasets underscores the reliability of the top‐performing algorithms. To further elucidate the performance of the algorithms, Table 11 is constructed. As shown in the table, the RBF and SVM models consistently outperformed other algorithms, achieving the lowest MAE and RMSE values, as well as the highest Kappa statistics and classification accuracy. These results highlight their robustness and reliability in classification tasks. In contrast, the Voted Perceptron and Simple Cart algorithms exhibited higher error rates, lower Kappa statistics and reduced classification accuracy, rendering them less suitable for this application. Although algorithms such as *K*NN and Random Forest offered a balance between accuracy and computational efficiency, the RBF and SVM models emerged as the most effective choices. These findings emphasize the importance of selecting appropriate algorithms to achieve optimal results in multiple birth data classification, with RBF and SVM being particularly well‐suited for this purpose.

**TABLE 11:** Evaluating of all algorithms based on various performance metrics.

	Mean absolute error		Root mean square		Kappa statistic (%)		Correctly classified instances % (value)		Incorrectly classified instances % (value)		Time taken (s)	
Algorithms	Train	Test	Train	Test	Train	30% test	Train	Test	Train	Test	Train	Test
MLP	0.0053	0.1877	0.0594	0.4194	0.9928	0.8475	99.6491	81.4208	0.3509	6.5745	63.49	63.74
RBF	0.0012	0.0186	0.0012	0.2313	1	0.6207	100	87.7049	0	18.5792	1.33	1.45
SVM	0.0015	0.1175	0.0342	0.3428	0.9976	0.7571	99.883	88.2514	0.117	11.7486	0.66	0.44
Logistic	0.0971	0.1198	0.2196	0.2806	0.8675	0.8168	93.5673	90.9836	6.4327	9.0164	0.73	0.39
Voted perceptron	0.3095	0.3451	0.5559	0.5869	0.3124	0.264	69.0058	65.5738	30.9942	34.4262	0.11	0.25
LWL	0.1546	0.1726	0.2663	0.3056	0.8298	0.7707	91.8129	88.7978	8.1871	11.2022	0	0
*K*NN	0.0012	0.0543	0.0208	0.2323	1	0.6775	100	84.153	0	15.847	0.02	0.08
Kstar	0.1537	0.1232	0.3703	0.2866	0.7068	0.0808	85.2459	84.0638	15.7664	17.9362	0	0
Bayes Net	0.048	0.1593	0.218	0.2364	0.9007	0.8884	95.2047	94.5355	4.7953	5.4645	0.13	0.24
Naïve Bayes	0.1009	0.0949	0.2968	0.2838	0.7892	0.804	89.9415	90.4372	10.0585	9.5628	0.05	0.09
AD Tree	0.123	0.134	0.2138	0.3976	0.8919	0.8623	94.7368	93.1694	5.2632	6.8306	0.2	0.14
BF Tree	0.0565	0.0945	0.1681	0.2587	0.9326	0.8455	96.7251	92.3497	3.2749	7.6503	2.01	0.45
Decision Stump	0.2254	0.2091	0.3357	0.3199	0.701	0.7397	85.9649	87.4317	14.0351	12.5683	0.08	0.06
FT	0.0254	0.0989	0.1343	0.3008	0.9664	0.8014	98.3626	90.1639	1.6374	9.8361	0.59	0.72
J48	0.0534	0.1036	0.1634	0.2744	0.9374	0.8234	96.9591	91.2568	3.0409	8.7432	0.25	0.37
LAD Tree	0.1549	0.1588	0.2736	0.2847	0.8378	0.8219	92.1637	91.2568	7.8363	8.7432	0.47	0.64
LMT	0.1229	0.1357	0.2327	0.2619	0.8595	0.8055	93.2164	90.4372	6.7836	9.5628	6.4	8.46
NB Tree	0.0235	0.0746	0.1362	0.2519	0.9519	0.8559	97.6608	92.8962	2.3392	7.1038	3.64	3.84
Random Forest	0.0536	0.1383	0.1079	0.2635	0.988	0.8942	99.4152	88.2514	0.5848	5.1913	0.2	0.13
Random Tree	0.125	0.1036	0.3155	0.2784	0.7958	0.8332	90.0585	91.8033	9.9415	8.1967	0.01	0.03
REP Tree	0.079	0.0907	0.1988	0.217	0.891	0.8829	94.7368	94.2623	5.2632	5.7377	0.10	0.14
Simple Cart	0.5559	0.1016	0.1883	0.2522	0.9182	0.8401	96.0234	92.0765	3.9766	7.9235	1.14	0.36

Abbreviations: *K*NN, *K*‐nearest neighbour; MLP, Multilayer Perceptron; RBF, Radial Basis Function; SVM, support vector machine.

Data mining has proven to be an invaluable tool in dairy cattle farming, enabling the analysis of critical factors influencing multiple birth rates, such as genetic composition, nutritional intake and environmental conditions. By leveraging historical data and advanced predictive models, farmers can make informed decisions to enhance the likelihood of multiple birth within their herds. The RBF and SVM algorithms, which demonstrated exceptional performance with low error rates and high classification accuracy, facilitate precise predictions of multiple birth events. Such predictive capabilities are crucial for targeted genetic selection and optimized resource allocation, ensuring that cows expected to produce multiple birth receive optimal care and nutrition. Moreover, accurate multiple birth predictions improve prenatal and postnatal management, thereby enhancing animal health and welfare while reducing the risk of complications. The economic implications of these advancements are profound, as refined breeding and management strategies can lead to increased productivity and higher financial returns. Identifying genetic lines with high multiple birth tendencies allows for selective breeding practices, further elevating the chances of multiple birth and contributing to herd development. Although the specific applications of classification systems in dairy cattle management are still emerging, their potential benefits are substantial. Implementing these systems can significantly enhance strategies aimed at improving milk yield per cow and maintaining ‘multiple birth‐friendliness’, thereby promoting both productivity and animal welfare. In conclusion, the integration of advanced classification methods into dairy cattle production fosters more efficient and sustainable agricultural practices, paving the way for more profitable and welfare‐conscious farming operations.

## 5 Conclusion

This study evaluated the efficacy of various algorithms in classifying multiple birth occurrences in dairy cattle, with a focus on metrics such as sensitivity, specificity, MAE and RMSE. The results demonstrated that the RBF and SVM models consistently outperformed other algorithms, achieving higher accuracy, lower error rates and greater classification reliability. These findings underscore their potential for accurately predicting multiple birth events, which is essential for targeted genetic selection and improved herd management practices. The predictive capabilities of the RBF and SVM models can inform selective breeding programmes, optimize resource allocation and enhance prenatal and postnatal care for cows expected to produce multiple birth. These advancements not only improve animal health and welfare but also yield significant economic benefits, highlighting the value of incorporating advanced classification techniques into dairy farming operations. This research underscores the importance of selecting appropriate algorithms to address specific challenges in agricultural data analysis. By leveraging predictive modelling, farmers can refine their breeding and management strategies, ultimately fostering increased productivity, sustainability and a focus on animal welfare. Future studies should explore additional applications of these methodologies to further enhance efficiency and profitability in dairy cattle production.

## Author Contributions

**Mostafa Ghaderi‐Zefrehei**: writing – original draft, investigation, formal analysis, conceptualization. **Maryam Montazeri‐Najafabadi**: methodology, resources. **Farjad Rafeie**: writing – review and editing, investigation. Effat Nasre Esfahani: writing – original draft, resources. **Mohammad Reza Abbaszadeh** Bavi Soflaei: methodology, formal analysis. **Mojtaba Kafi**: writing – review and editing. **Mohammad Hosein Banabazi**: investigation, conceptualization, writing – review and editing.

## Funding

The authors have nothing to report.

## Conflicts of Interest

The authors declare no conflicts of interest.

## Data Availability

The data that support the findings of this study are available from the corresponding authors upon reasonable request.
